# High-Capacity, Fast-Response, and Photocapacitor-Based Terpolymer Phosphor Composite

**DOI:** 10.3390/polym12020349

**Published:** 2020-02-06

**Authors:** Marwa Mokni, Francesco Pedroli, Giulia D’Ambrogio, Minh-Quyen Le, Pierre-Jean Cottinet, Jean-Fabien Capsal

**Affiliations:** Electrical Department, University Lyon, INSA-Lyon, Ladoua Campus, LGEF Loboratory, EA682, F-69621 Villeurbanne, France; mokni.marwa88@gmail.com (M.M.); francesco.pedroli@insa-lyon.fr (F.P.); giulia.dambrogio@insa-lyon.fr (G.D.); minh-quyen.le@insa-lyon.fr (M.-Q.L.); pierre-jean.cottinet@insa-lyon.fr (P.-J.C.)

**Keywords:** photocapacitive detector, phosphor/terpolymer composite, photoelectrical conversion, light sensor, artificial retinal prosthetic device

## Abstract

This paper describes a new class of light transducer-based poly (vinylidene fluoride-trifluoroethylene-chlorotrifluoroethylene) (P(VDF-TrFE-CTFE)) terpolymer doped with 50% wt. phosphor particles that enables to efficiently transform light energy into an electrical signal. Broadband dielectric characterization together with experimental results on photo-electric conversion demonstrated high capacitance variation of the proposed composite under light exposure, confirming promising potential of our sensor device for application in retinal prostheses where the converted electrical signal can affect the biological activity of the neuron system. In addition to the benefit of being light-weight, having ultra-flexibility, and used in a simple process, the proposed photodetector composite leads to fast response and high sensibility in terms of photoelectrical coupling where significant increases in capacitance change of 78% and 25% have been recorded under blue and green light sources, respectively. These results demonstrated high-performance material design where phosphor filler contributes to promote charge-discharge efficiency as well as reduced dielectric loss in P(VDF-TrFE-CTFE), which facilitate the composite for flexible light transducer applications, especially in the medical environment.

## 1. Introduction

The emerging field of bioelectronics integration offers new physical development regarding neuroelectronic components [1]. The integration of malleable materials such as inorganic conductors and semiconductors including conjugated polymers remains attractive for neuronal applications [2,3,4] particularly for artificial retinal devices [5,6]. High flexibility and large mechanical deformation of the malleable host material is required for the practical application of bioelectronics in order to operate under humidity and biocompatible environments. Indeed, the use of light as a stimulation tool has emerged during the last decade as a valid alternative to electrical stimulation [7,8]. Light-sensitive materials depend on optimized illumination transducers that can convert luminosity to electrical stimuli that are then delivered to the retina. In the retina, light transmitted through lens in human eyes travels through several neuronal layers of the inner retina with virtually zero loss before reaching the photoreceptors. These photoreceptors efficiently absorb light and transform it into an electrical/biochemical signal to the bipolar cells [9]. For instance, in many blindness-causing diseases, photoreceptors degenerate, whereas second-order and projecting neurons are largely unaffected. Thus, the challenges of restoring vision in affected patients launched the development of artificial photoreceptors for retinal prosthetic devices, e.g., using light-directed electrical neuronal stimulation [10,11].

Several recent studies have sought novel materials with better properties for optical stimulation in terms of compactness, stability in physiological conditions, long-term biocompatibility, and high photo-electric coupling that is strong enough to electrically stimulate neurons with safe light intensities. For instance, the semiconductor layers developed by Rand et al. [12] are made using ubiquitous and nontoxic commercial pigments via simple and scalable deposition techniques. These devices are the first non-Si optoelectronic platform capable of sufficiently large photovoltages and displacement currents to enable true capacitive stimulation of excitable cells. Another technique based on fabrication of multiple silicon photovoltaic arrays [13] that were implanted subretinally via single retinotomy in rabbits. Optical coherence tomography confirmed complete reattachment of the retina over the implants. Alternatively, Ghezzi et al. [14] proposed a hybrid bioorganic system by interfacing an organic semiconductor with a network of cultured primary neurons, which can be successfully grown onto the polymer layer without affecting the optoelectronic properties of the active material or the biological functionality of neuronal network. Antognazza et al. [15] characterized biophysical and surface properties of an organic device based 3,4-poly(ethylenedioxythiophene):polystyrenesulfonate (PEDOT:PSS) material, following by an analysis of long-term biocompatibility after implantation of the organic device in subretinal space of rats for a period of five months. The results indicate a good stability of the subretinal implants over time.

Concerning the shape of the photo-transducers, it varies from planar interfaces to micro-structured surfaces and nano- or micro-particles. In particular, electrical fields can be exploited to locally stimulate cells and tissue, inducing different responses, for instance, altering the electrophysiological activity of electrogenic cells or modulating certain cellular processes and functionalities [16]. Recently, silicon-based devices are widely employed for electrical interfaces both at the micro and nanometer scale with neural tissues [17] and photovoltaic-based platforms for retinal implants. The effects of this interface on cells can be investigated by observing capacitive, chemical, and thermal mechanisms involving the cell membrane. In particular, platforms based on electrical photo-transduction have aroused the interest of many research groups with the purpose of recording and stimulating single cells, cell networks, or tissues [18].

The conversion of light into electrical signals is a critical function of the retina, and thus light-sensitive materials are widely considered for artificial retina. Based on this observation, we propose an ultra-flexible and high-performance photodetector based on a light-sensitive composite material that ensures the dispersion of photoluminescent particles into a poly (vinylidene fluoride-trifluoroethylene-chlorotrifluoroethylene) P(VDF-TrFE-CTFE) terpolymer matrix. As it was demonstrated in [19], the P(VDF-TrFE-CFE) terpolymer exhibited significantly higher dielectric constant (i.e., the permittivity) with respected to the conventional electroactive polymers and the PVDF-based copolymer. The study reported in [3,4] confirmed that the mechanical strain of the terpolymer can be highly enhanced by increasing its dielectric property, although the material is applied under very low electric field. This makes a terpolymer as one of the most appropriate candidate for various applications in actuating/sensing devices where high flexibility is primordial. Among the currently available commercialized particles, phosphor has been chosen due to its cost effectiveness and its multifunctional optical materials, e.g., light emitting in the visible spectrum. Furthermore, phosphor is one of the most suitable materials for use as information display screens because of its good definition and brightness characteristics. Note that while light can be converted into electrical signals, electrical signals can also be converted into light, making it possible to exploit phosphor as either a light emitter or photodetector. To some extent, when this material is subjected to an alternating current field, it can emit visible radiation whose intensity is a function of the applied voltage as well as the frequency. On the other hand, its electrical properties can be changed when it is photo-excited by exposure to a sufficiently intense light source. In this study, we only investigate the second effect of the particulate polymer composites embedded with phosphors that become self-sensing structures [20]. Depositing directly photoluminescent particles onto electroactive materials has recently propelled the growth of printable electronic devices. A benefit of using composite architecture is its unique combination of low density together with multifunctionalities, which can be leveraged to produce advanced technologies [21,22] in various actuating/sensing applications.

## 2. Materials and Methods

### 2.1. Polymer Fabrication

The new light polymeric composite photoreceiver was prepared using a solution casting method. The commercial fluorinated terpolymer powder (purchased from Piezotech S.A.S, Arkema group, Lyon, France) P(VDF-TrFE-CFE) 56.2%–36.3%–7.5% [23] was dissolved with butanone, also known as methyl ethyl ketone (MEK) in a fraction of 25% wt. When the solution achieved a homogenous state, Phosphor powder (GG45) was filled into the terpolymer solution with a content of 50% wt. This ratio was chosen for achieving the best trade-off between the photocapacitive performance and the dispersion of the sample. Indeed, higher phosphor concentration led to enhanced dielectric property of the capacitor, but this parameter should be limited in order not to much influence on the homogeneity as well as the flexibility of the composite. The prepared mixture was then casted on a conductive thin ITO/PET substrate of 2-µm thickness (Indium Tin Oxide coated on transparent Polyethylene Terephthalate surface), which was purchased from Thorlabs company (Newton, NJ, USA). The ITO-coated PET films was chosen due to their high conductivity and optical transmission in large spectral range (400 nm–2110 nm). Furthermore, this material is very flexible and high temperature resistant up to 120 °C.

**Figure 1:**
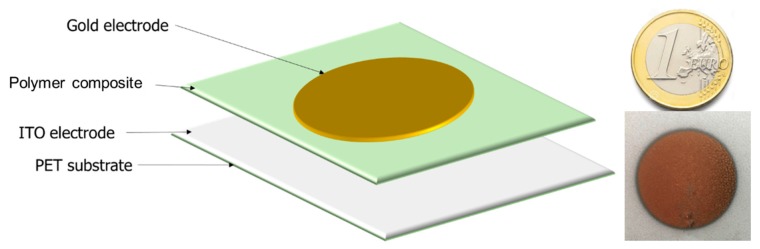
Structure of a flexible terpolymer/phosphor composite.

The first thermal treatment of the films was performed at 70 °C for 10 min to evaporate the solvent. Subsequently, it was annealed at 100 °C for 1 h in a convection oven to promote terpolymer crystallization [24]. For photo-capacitor characterization of the polymer composite, samples were sputtered on the top with a 20-mm diameter circular gold electrode using a Cressington Sputter Coater (208 HR) that helps to deposit a very thin (25 nm) Au layer. On the other hand, for the bottom electrode, the 100 µm-thick conductive substrate ITO/PET was used because of its flexibility and transparency to visible light. The thickness of all samples was around 150 µm. The architecture of a flexible terpolymer/phosphor is illustrated in Figure 1.

### 2.2. Experimental Setup and Characterization Methods

**Figure 2:**
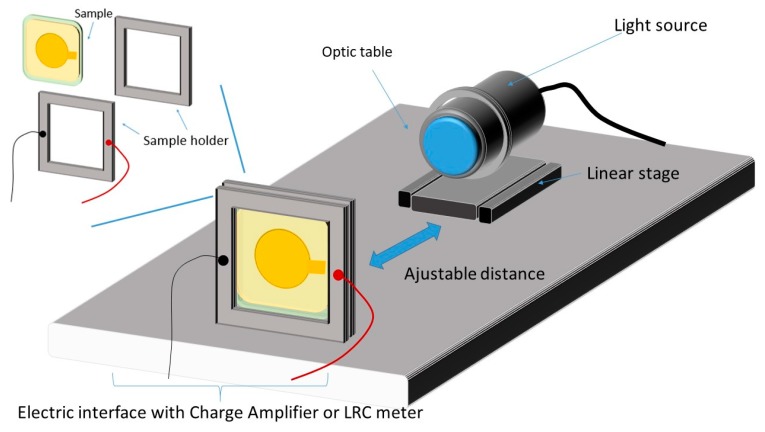
Experimental setup for photo-capacitor characterization of the polymer composite.

To assess the performance of the photo-capacitor sensing ability and the dielectric properties of the developed phosphor/terpolymer composite, a dedicated experimental test was carried out as depicted in Figure 2. The fabricated sample was excited by different wavelength light sources (Thorlabs, Newton, NJ, USA) [25] corresponding to three colors (red, green, and blue) whose features are described in Table 1. These LED array sources can be mounted in a 30-mm support system consisting of two moving cage plates that allow to clamp the LED in place for perfectly horizontal light emission. An optical chopper device (MC2000B controller) including various wheels create a system, and large frequency ranges (from 4 Hz to 10 kHz) were investigated in order to generate an alternative pulse with very highly accurate frequency.

Regarding the photo-electric coupling characterization, the resulting current delivered by the photo-capacitive material powered by an input light source was empirically measured via a low-noise current preamplifier (Stanford, SR570 Model). Additionally, the charge displacement was detected using a high-precision charge amplifier (KISTLER type 5015 Instruments, Winterthur, Swiss). Finally, all signals including electrical charge, light intensity, and output current were simultaneously recorded in real-time using a Sirus 8XSGT card (DEWESoft France, Ivry-sur-Seine, France). Post-data treatment was performed using Origin software (Originlab Corporation, WA, USA).

Concerning the electrical characterization, dielectric measurements at a wide frequency range of 0.1 Hz to 1 MHz were carried out at room temperature using a Solartron (SI 1255) impedance meter. In order to understand the influence of light (e.g., wavelength, intensity) on the capacitance change of the proposed material, measurements based on an LCR meter (Keysight E4980A, Santa Rosa, CA, USA) under an alternative input voltage of 1 V and 1 kHz were taken.

## 3. Results and Discussion

**Figure 3:**
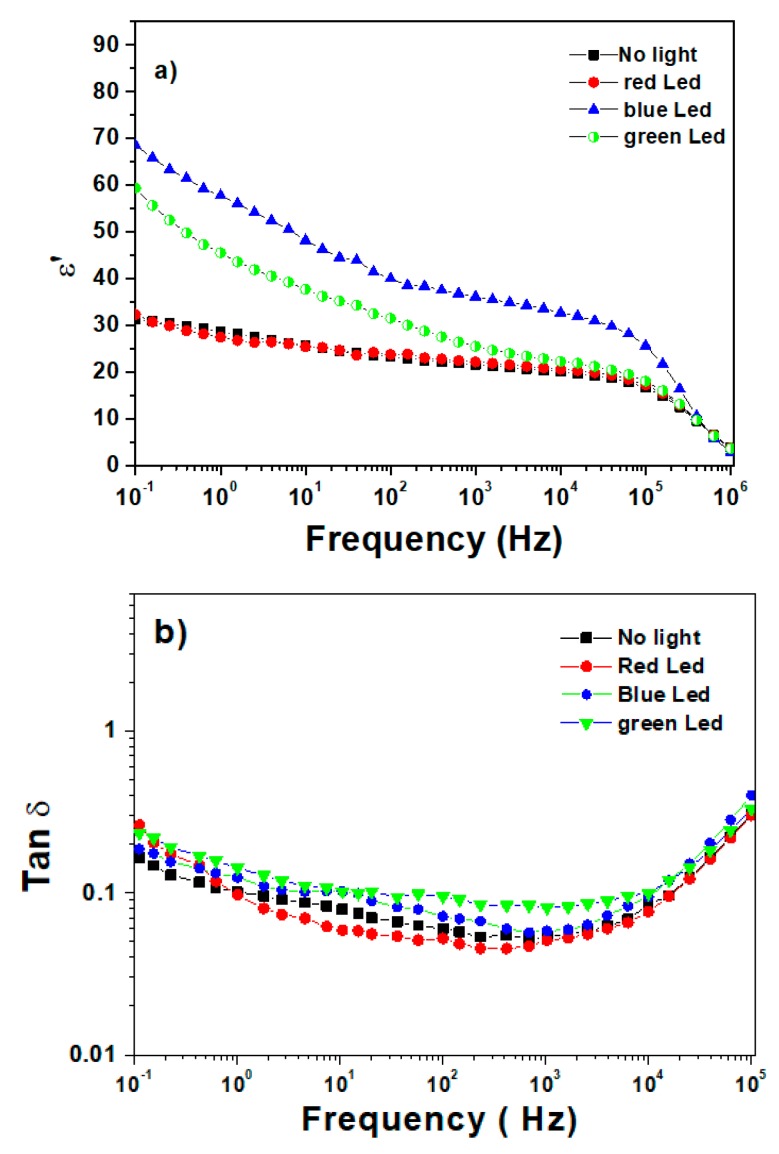
Frequency dependence at various LED lights of (**a**) the real part of complex permittivity and (**b**) the dissipation factor.

The evolution of dielectric properties comprising relative permittivity and tanδ losses versus frequency in a dark room as well as in three different light wavelengths (red, green, blue) is shown in Figure 3a and Figure 3b, respectively. Under low frequency (i.e., 0.1–1 Hz), the blue and green LEDs generated a two-fold increase in dielectric permittivity of the composite, which is contrary to the case with the red LED where no enhancement in electrical properties were achieved. This can be explained by the fact that the phosphor composite is more sensitive to the blue and green illumination under which light energy can convert to electrical energy. Concerning the dielectric losses, tanδ, on the other hand, remained quasi-constant over a large frequency range regardless of the light excitation. Such a property indicates a purely capacitive effect of the polymeric sensor induced by the light response of the phosphor composite.

**Figure 4:**
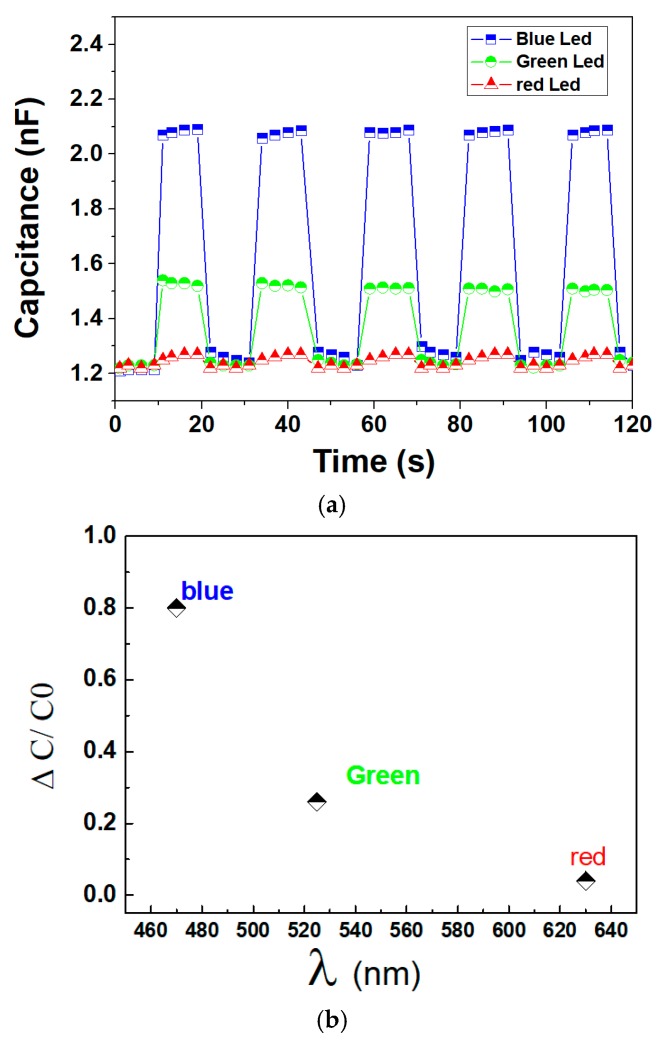
(**a**) Capacitance of polymer composite excited by blue and green lights with on/off cycles. (**b**) Capacitance change (Δ*C*/C_0_) under different wavelength exposure, where Δ*C* = C − C_0_; C and C_0_ are, respectively, the capacitance value of the composite with and without light application.

To highlight the dielectric enhancement of the developed photosensor, the time evolution of the capacitance under an alternative ON/OFF light source was carried out. As expected, in Figure 4a, high variation in capacitance was recorded with an increase of 78% and 25% when the P(VDF-TrFE-CTFE) composite was illuminated by the blue and the green LEDs, respectively. Oppositely, the red LED results in insignificant capacitance change of the material, which is in accordance with its dielectric behavior analyzed previously. It is noteworthy that under the light ON or light OFF states, the capacitance remained constant, confirming satisfactory reproducibility and homogeneity of the photo-receiver based phosphor/terpolymer matrix. Figure 4b illustrates the relative value of the capacitance change Δ*C*/*C*_0_ as a function of the wavelength. It is obvious that in our system, the selected dopant particle as photoluminescent phosphor (GG45) shows the highest absorbance to blue light, and the sensitivity drastically enhances the order of red, green, and blue. Accordingly, Figure 4 demonstrates that our proposed photodetector exhibits a stable electrical response as well as fast capacitance change with the varying light intensity, showing widespread potential of such materials for various applications of photo-sensing device with excellent precision and sensitivity.

**Figure 5:**
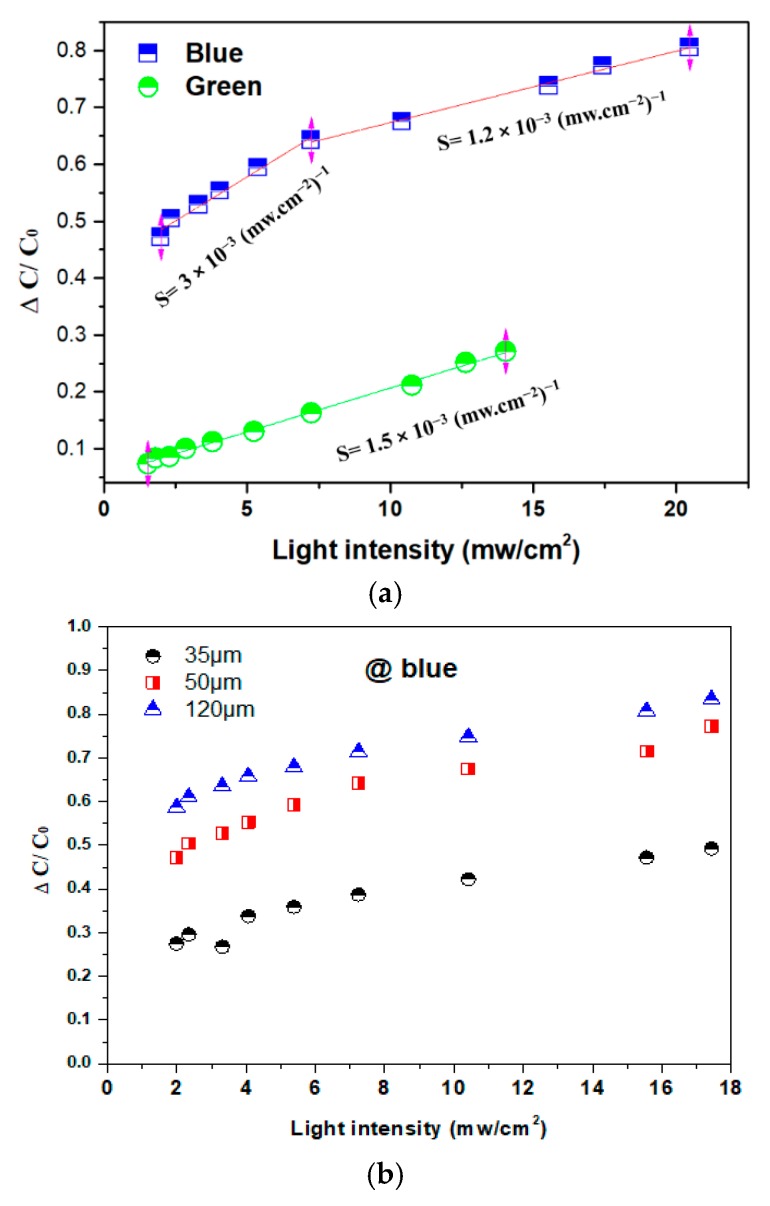
(**a**) Capacitance change versus light intensity with: (**a**) blue and green LEDs and, (**b**) only blue LED with different thicknesses of polymer composites.

Figure 5a illustrates the linear relationship between the relative capacitance variation of the terpolymer composite versus the light intensity of 20 mW/cm^2^ and 14 mW/cm^2^ delivered by the blue and the green LEDs, respectively. For a given power intensity, the sample treated under the blue source exhibits around four-times higher capacitance variation as opposed to the case with the green source. This result, again, confirms that the photoluminescent phosphor is capable to absorb more efficiently the blue wavelength rather than the green one, resulting in better photo-electric energy conversion. To better assess the photocapacitive transduction properties of the composite, the sensitivity *S* [26] was graphically determined as the slope of the capacitance/intensity curve in Figure 5a. Interestingly, the green LED leads to a constant sensitivity of 1.5 W^−1^·cm^2^ for a whole range of intensity, whereas in the case of the blue LED, the terpolymer composite exhibits two different levels of sensitivity, i.e., under low power density less than 7 mW/cm^2^, *S* = 3 W/cm^2^, which is double that of the green LED. Under high power density (7 mW/cm^2^ ≤ I ≤ 20 mW/cm^2^), the sensitivity *S* radically reduces and attains the value similar to the case with the green LED. Overall, the developed photocapacitor leads to higher sensitivity with the blue light but seems to be more stable with the green light. Indeed, the sensitivity as a function of light intensity and wavelength strongly depends on the dopant composition, concentration, and required excitation energy [27]. The results reveal the importance of photocapacitive material selection for the final application dedicated to the sensing device. For instance, in the area of retinal implants, it has been reported in [5] that human retinas are very sensitive to green light. Furthermore, compared to blue light, green light is less intensive and therefore safer for retinal prosthesis, which can be damaged by short wavelengths (particularly those less than 600 nm) for exposures longer than 1 s. Based on the compromises of retina safety as well as the level and stability of the material sensitivity, the design of the photocapacitve sensor based on green light excitation has been demonstrated to be more effective.

Figure 5b shows the evolution of the capacitance variation for three samples with different thicknesses as a function of the blue light intensity. The result demonstrates an improvement of the relative capacitance change with an increase in the thickness of the film, showing that the dielectric properties of the photo-composite are not only dependent on the power density and the wavelength of light but also on its geometry. To some extent, higher thickness somewhat favors the photoelectric conversion that intrinsically occurs inside the photoluminescent phosphor particles. These characteristics should be taken into account on the material design for better optimizing its sensing performance.

**Figure 6:**
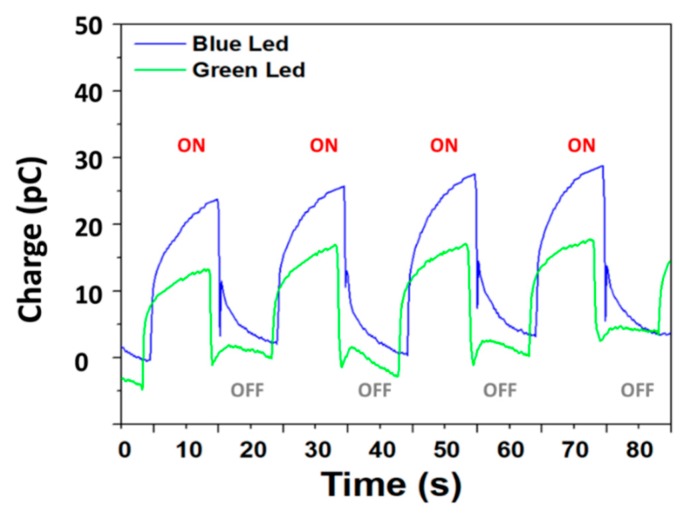
Charge–discharge behavior of terpolymer phosphor composite excited under blue and green LEDs.

The charges generated by the composite sample were measured with blue and green luminescence are presented in Figure 6 and display the charge and discharge behavior of the photo-composite under a 50-mHz alternative on/off blue and green light. As shown, the photo-charge generated by a pulse illumination for 10 s makes it possible to create accumulated charges of 25 pC and 14 pC when applied by the blue and the green LEDs, respectively. Recently, Abdullaeva et al. [28] assumed that during the pulse stimulation there is an accumulation of negative charge carriers at the photoconductor–electrolyte interface, and this effect leads to cell depolarization and rapid hyperpolarization of the cell membrane.

**Figure 7:**
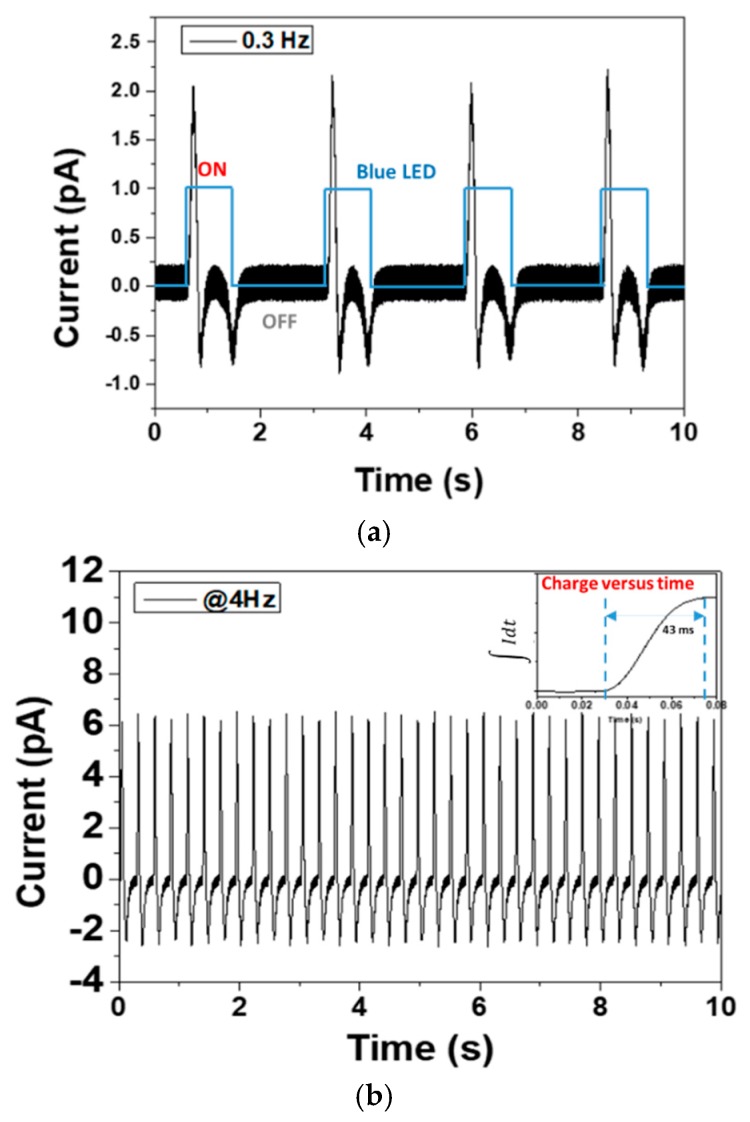
Photocurrent at various frequencies: (**a**) 0.3 Hz, (**b**) 4 Hz, Inset: charge displacement versus time.

Figure 7 displays the delivered current response under different dynamics of alternative blue LEDs that correspond to 0.3 Hz (a) and 4 Hz (b) at similar light intensity of 15.5 mW/cm^2^. During the ON state, the transient current positively increases very quickly to fully charge the photocapacitor and then rapidly drops to a negative value with an important overshoot, showing an exponential decay that match the characteristics of double-layer capacitors under the constant-voltage mode [29]. The transient photocurrent behavior is probably due to a generation of charge carriers form a space-charge region within the semiconductor accumulated at the phosphor/polymer interface [30]. This in turn can be affected by numerous parameters like thickness of the photoactive layer, direction of illumination [31], excitation wavelength, particle content, and choice of electrode and electrolyte or solid dielectric layer. After the transient state, the steady state is established, allowing the current to progressively increase to attain the zero level. On the other hand, during the OFF state, the transient current shows a reversed effect with negative polarity, as observed in Figure 7a. Interestingly, such a behavior was not clearly shown under higher frequency light of 4 Hz (cf. Figure 7b), where only one negative peak has been found. This property should be related to the intrinsic electrical relaxation of the material versus the applied photo-dynamic excitation.

In conclusion, during each period of the ON/OFF cycle, the photocurrent exhibited two principal transient peaks with opposite polarity, which is similar to the result reported by Reissig et al. [24] where they also found a double peak of the output delivered by a P3HT:PCBM blend filled with an ionic liquid. In this case, the charge attribution may occur by accumulated charge carriers and accumulation of holes inside the material matrix.

It is noteworthy that higher stimulation frequency (i.e., 4 Hz) leads to a faster response as well as a 3-fold increase in current intensity compared to that at a stimulation frequency of 0.3 Hz. As illustrated in the small inset at the top right corner of Figure 7b, the necessary time for the charge displacement attains its final target value at about 43 ms, which is faster compared to Photodetector P(8-AZO-10) investigated in [32] (60 ms) under a similar input application. Therefore, our developed composite seems to be a very promising candidate for human retinal prosthesis whose response time requirement should not be less than 70 ms.

**Figure 8:**
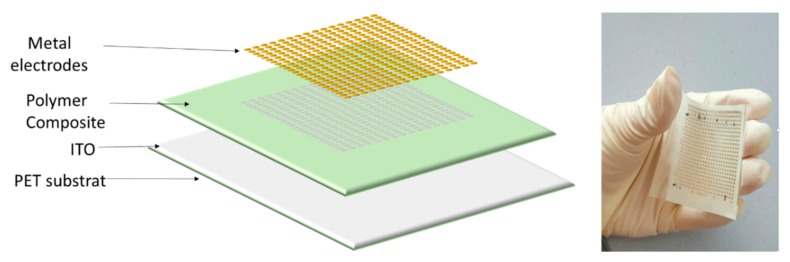
Flexible photodetector arrays, consisting of Indium Tin Oxide (ITO)-coated Polyethylene Terephthalate (PET)/polymer composite/Au electrode.

To better verify the electrical-response performance of the material under an excited illumination, a flexible capacitive matrix-type photodetector was investigated, as described in Figure 8. A 50 × 50 mm^2^ phosphor/terpolymer composite was sandwiched between conductive ITO-coated PET substrate and 25 × 25 gold electrodes in a 1-mm diameter circle to create a multipoint light distribution applied on the sample surface. The total thickness of the structure is around 200 µm.

**Figure 9:**
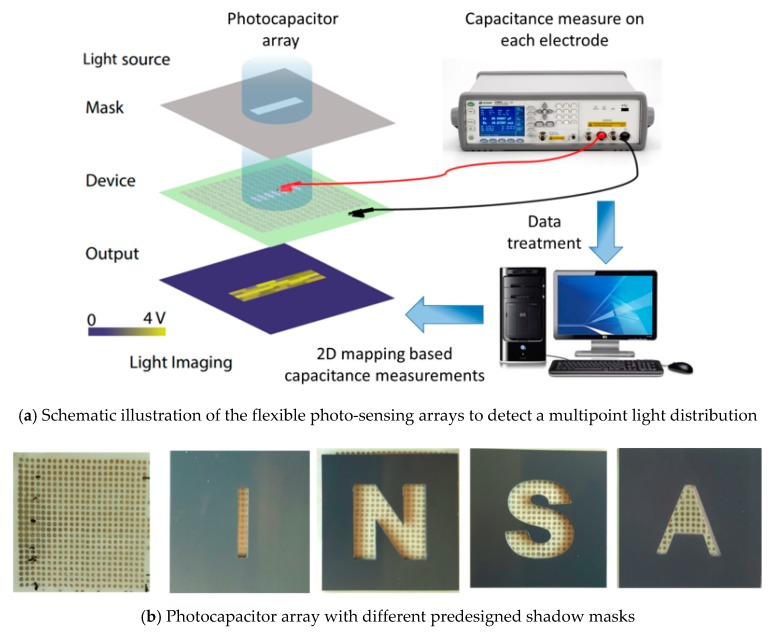
Photo-capacitor arrays using letter-shaped masks.

**Figure 10:**
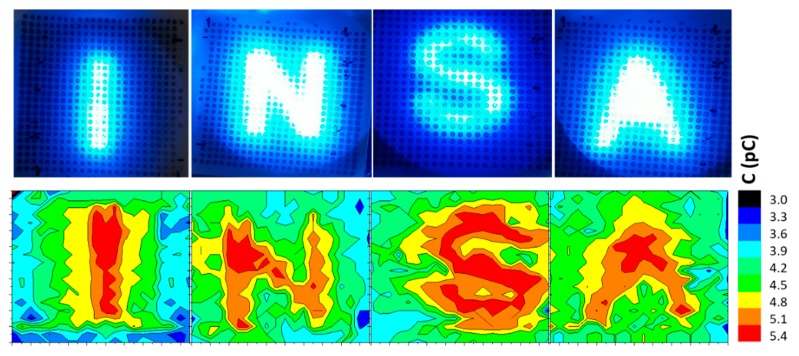
Schematic illustration corresponding to the capacitance measurement of flexible photocapacitor arrays.

As shown in Figure 9a, a pre-designed shadow mask was placed between the blue LED source (16 mW/cm^2^) and the photo-sensing device to form a small light spot. The proposed technique would permit reliable retinal stimulation with a small surface of multiple electrodes [9], on which current density and charge density increase rapidly. An empirical test was carried out using four different letter-shaped masks “INSA”, as represented on Figure 9b. The capacitance of each illuminated electrode point was determined via a high-accuracy impedance meter (Keysight E4980A) under a dynamic of 1 kHz. All data were then plotted using ORIGIN software. A 2D mapping corresponding to the 25 × 25 capacitance measurements of the designed photodetector is illustrated in Figure 10, where a scale of the capacitance value from 3 pC to 5.4 pC was transformed to the color scale. It is clear that active sensing of the area of the letter pattern with blue illumination had higher capacitance values, while others that were blocked by the designed mask had no obvious change in the dielectric property. Four clear letters “INSA” can be identified in the mapping results (Figure 10), indicating excellent photoelectrical conversion of our flexible sensor array. Finally, these experimental results seem to be very promising, demonstrating a feasibility of the developed composite for several applications, including photodetectors and retinal prostheses.

## 4. Conclusions

In this work, we have introduced flexible photodetector arrays based on a simple fabrication process of the terpolymer P(VDF-TrFE-CTFE) filled with photoluminescent phosphor particles. The broadband dielectric spectroscopy analysis showed high sensitivity of the capacitive ability versus blue and green lights without degrading its dielectric losses. Contrary to the case with the red light, no enhancement in electrical properties were achieved, which was probably due to the fact that the phosphor composite is more sensitive to the blue and green illuminations. It was revealed that the design of the photocapacitve sensor showed the highest absorbance to the blue light excitation, but the green one has been demonstrated to be more effective regarding retina safety and stability of the material sensitivity. Most importantly, experimental results demonstrated a possibility to attain fast photoelectrical response (i.e., 43 ms), which is one of pertinent factors in application of artificial retinal implants. In conclusion, the fabricated composite seems to be an appropriate candidate to efficiently convert light to electric signals, making it possible to achieve high sensitivity flexible photosensor for recent neuroprothetic development on new vision restoration. In future studies, further investigations will seek to enhance variation of electric parameters induced by the light-sensor mode as well as the biocompatibility of adhesion layers in order to meet medical requirements.

## Figures and Tables

**Table 1:** Properties of Thorlabs’ LED array light sources [5], including 3 different colors Blue, Green, and Red, that was specified by the wavelength.

Reference	LIU470A	LIU525B	LIU630A
LED’s Color	Blue	Green	Red
Wavelength (nm)	470	525	590
Intensity at a distance of 100 mm (mW/cm^2^)	4.0	1.9	2.4
Total output power (mW)	253	111	208
